# Supplementary material for: Generation of third-harmonic spin oscillation from strong spin precession induced by terahertz magnetic near fields
Source: Nat Commun. 2023 Mar 31;14:1795. doi: 10.1038/s41467-023-37473-1 (PMC10066181; doi:10.1038/s41467-023-37473-1)
Supplement: Supplementary file 1 — Supplementary Information [file 41467_2023_37473_MOESM1_ESM.pdf]

Supplementary information for

Generation of third-harmonic spin oscillation from strong spin precession induced by terahertz magnetic near fields

Zhenya Zhang[1], Fumiya Sekiguchi[1], Takahiro Moriyama[1], Shunsuke C. Furuya[2], Masahiro Sato[3], Takuya Satoh[4], Yu Mukai[5], Koichiro Tanaka[6], Takafumi Yamamoto[7], Hiroshi Kageyama[8], Yoshihiko Kanemitsu[1], and Hideki Hirori[1]

[1]*Institute for Chemical Research, Kyoto University, Uji, Kyoto 611-0011, Japan*

[2]*Department of Basic Science, University of Tokyo, Meguro, Tokyo 153-8902, Japan*

[3]*Department of Physics, Chiba University, Chiba 263-8522, Japan*

[4]*Department of Physics, Tokyo Institute of Technology, Tokyo 152-8551, Japan*

[5]*Department of Electronic Science and Engineering, Kyoto University, Kyoto, Kyoto 615-8510, Japan*

[6]*Department of Physics, Graduate School of Science, Kyoto University, Kyoto, Kyoto 606-8502, Japan*

[7]*Laboratory for Materials and Structures, Tokyo Institute of Technology, Yokohama, Kanagawa 226-8503, Japan*

[8]*Department of Energy and Hydrocarbon Chemistry, Graduate School of Engineering, Kyoto University, Kyoto, Kyoto 615-8510, Japan*

I. Experimental setup

The setup described in the Method section of the main text is shown in Fig. S1. An amplified Ti:sapphire laser (repetition rate 1 kHz, central wavelength 800 nm, pulse duration 80 fs, and 7 mJ/pulse) was used to generate intense terahertz (THz) pulses by optical rectification of the near-infrared (NIR) femtosecond laser pulses (Fig. S1; red beam) in a $LiNbO_3$ crystal by using the tilted-pump-pulse-front scheme. The THz pulses (Fig. S1; grey beam) were focused on the gold microstructure by using an off-axis parabolic mirror with a focal length of 50 mm, resulting in a spot diameter of ~300 μm (full width at half-maximum). The direction of the THz electric field after the $LiNbO_3$ crystal and after the wire-grid pair is normal to the paper (as indicated by the vector $\boldsymbol{E}_{\mathrm{THz}}$ below the low-pass filter). The pair of wire grids is used to tune the amplitude of the THz field. The NIR probe pulses (with a linear polarization normal to the paper as indicated by the vector $\boldsymbol{E}_{\mathrm{probe}}$ above the beam splitter) were focused on the sample surface by a 50× objective lens, resulting in a spot diameter of ~1.2 μm. The probe pulses propagated through the sample and then were reflected at the surface as shown in the inset (a). The ellipticity angle η of the probe light was decoded by a λ/4 plate, a Wollaston prism and a balanced detector.

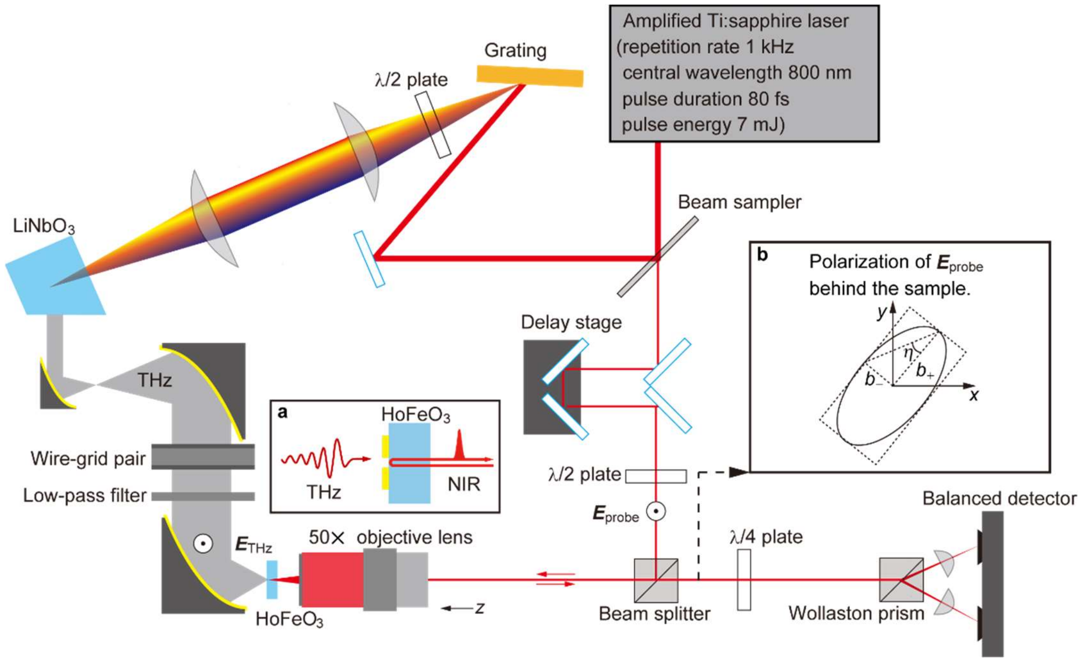

Figure S1. Schematic of the setup for the time-resolved measurement of η. The inset (a) shows the process of the transmission and reflection of the NIR probe light in the sample. The inset (b) shows the polarization ellipse of the NIR probe light after the propagation through the sample. The ellipticity angle η is defined by the inverse tangent of the ratio of the minor axis to the major axis of the ellipse, b_- and b_+, respectively.

The hysteresis data of $HoFeO_3$ at room temperature is shown in Fig. S2. As shown by the hysteresis loop at room temperature in Fig. S2, the static magnetic field required to reverse the magnetization is ~50 mT, which is common for RFeO$_3$ (R is a rare-earth element). Regarding the experiment with the THz pulse, we need to consider that the THz magnetic field is a transient pulse with a duration on the order of picoseconds. In contrast to the result for the static magnetic field, the coherent reversal of magnetization is much more difficult in the case of such a short pulse.

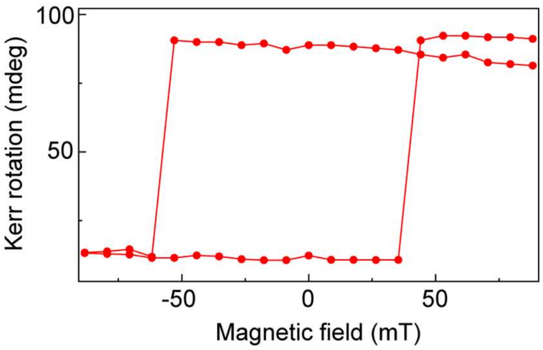

Figure S2. Magnetic field dependence of the Kerr rotation of $HoFeO_3$ at room temperature. The probe wavelength is 633 nm.

II. Calculation of magnetic and electric THz near-field

The magnetic near-field generated in the spiral of the microstructure is calculated by a finite-difference time-domain (FDTD) simulation as shown in Figs. S3 and S4. The variable z describes the depth: the $HoFeO_3$ surface with the microstructure is located at $z = 0$ and $z < 0$ corresponds to positions inside the crystal as shown in Fig. 1 of the main text. Here, the x- and y-axes components of the magnetic near-field are ignored, because only the z-axis component can excite the quasi antiferromagnetic (q-AF) mode at 0.58 THz. The input THz pulse used for the FDTD simulation is shown by the black curve in Fig. 1b in the main text. The results of $\boldsymbol{B}(0, t)$ and $\boldsymbol{E}(0, t)$ are shown in Fig. S3. The z dependence of the peak amplitude of the magnetic near-field, $B_{z,\text{peak}}$, is shown in Fig. S4.

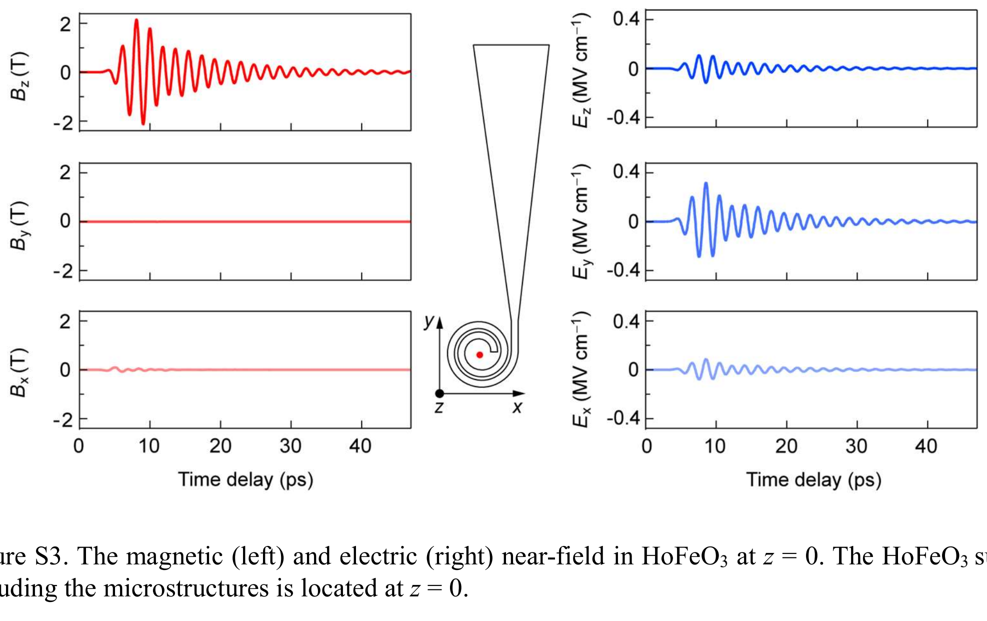

Figure S3. The magnetic (left) and electric (right) near-field in $HoFeO_3$ at $z = 0$. The $HoFeO_3$ surface including the microstructures is located at $z = 0$.

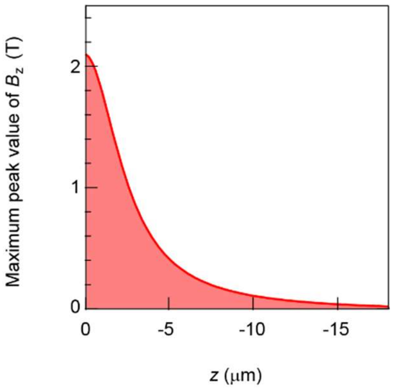

Figure S4. Depth dependence of the maximum peak amplitude of the magnetic near-field in $HoFeO_3$. The $HoFeO_3$ surface including the microstructures is located at $z = 0$.

III. Electric field at the tip of the spiral and no sample damage

Regarding the maximum electric field, the enhancement of the electric field is the strongest near the tip of the spiral as shown in the Fig. S5a. The magnitudes of the electric field at the red and blue circle are shown in the Figs. S5b and c; the peak amplitudes are ~7 MV cm^{-1} and ~4 MV cm^{-1}, respectively. However, the enhancement factor quickly becomes smaller at positions further away from the tip of the spiral, and the field becomes almost uniform near the center of the spiral.

We confirmed that no sample damage is induced by this electric field: In our experiment, the data of the initial measurement can be reproduced. This reproducibility of the data confirms that the sample around the probe position is not destroyed.

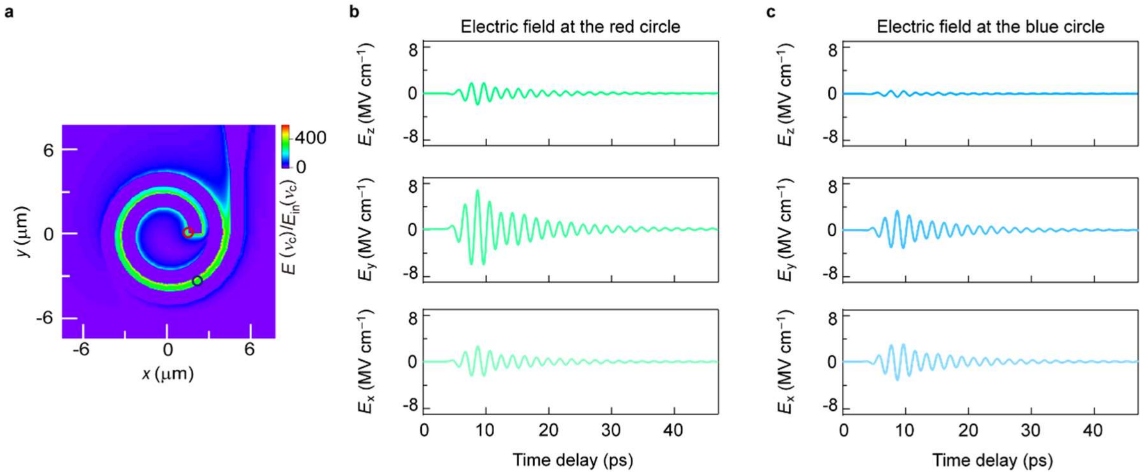

Figure S5. Distribution of the electric-field enhancement factor. (a) The spatial distribution of the electric field enhancement $E(\nu_c)/E_{in}(\nu_c)$. The temporal evolutions of the electric field along the x-, y-, and z-directions at the positions indicated by the red (b) and blue (c) circles in (a) are shown.

IV. Magnetic field and probe pulse distribution at the center of the spiral

Here, we would like to show that the magnetic field distribution is almost uniform in the probed region:

An image of the beam spot of the probe pulse is provided in Fig. S6a. The red dots in Fig. S6b, we show the cross section of the probe spot along the red dashed line. We fitted these red points to a Gaussian function and obtained a 1/*e*-diameter of 1.2 μm. Inside the 1/*e*-diameter, the distribution of the enhancement factor is almost uniform as shown by the blue curve in Fig. S6b. Therefore, it is reasonable to perform a calculation that ignores the magnetic field distribution in the *xy* plane.

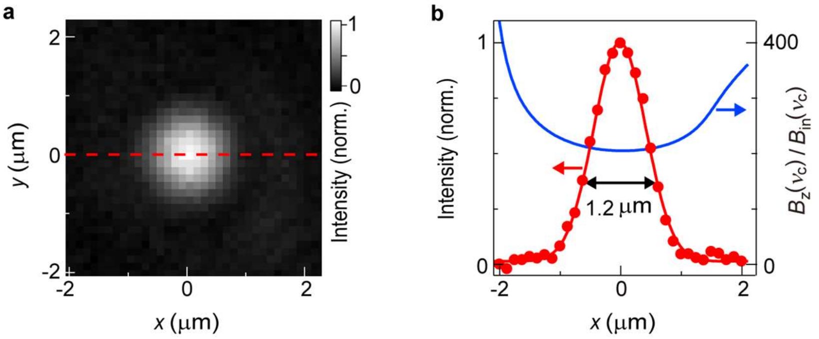

Figure S6. Probe pulse area. (a) The beam spot of the probe pulse. (b) The profile of the beam spot. The red points are the data along the red dashed line in a, and the red curve is a fit to a Gaussian function. The blue curve is the cross section of the bottom graph in Fig. 1c for $y = 0$ in the main text.

V. Influence of the electrical pulse on $\Delta\eta$

Fig. 2 in the main text ignoring offsets is shown in Fig. S7a for contrast. To investigate the influence of electric field, we performed another experiment as shown in Fig. S7b. As shown in Fig. S3 of the Supplementary Information, the electric field in the center of the microstructure is ~0.3 MV cm^{-1} along the *y* axis. In this experiment, a THz electric pulse polarized along the *y* axis with a peak amplitude of about 1 MV cm^{-1} was directly incident on a sample without microstructures. Considering the reflection loss, the electric field of this propagating THz pulse (not near field) in $HoFeO_3$ is 0.31 MV cm^{-1}. The result of the experiment is shown in Fig. S7b: no signal can be observed. This confirms that the demagnetization or spin rotation cannot be induced by the THz electric field in our experiment.

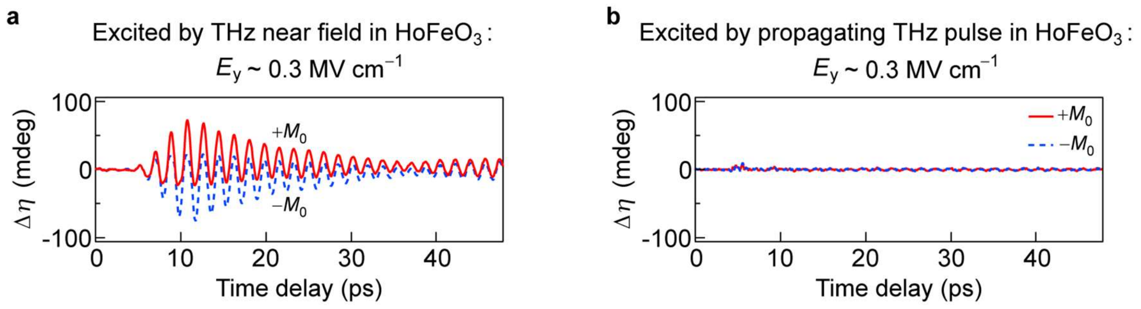

Figure S7. Comparison of $\Delta\eta$ between the excitations with THz near field and propagating THz pulse. The red and blue dashed curves are the ellipticity angle changes measured for initial magnetizations of $+M_0$ and $-M_0$. (a) The experiment data is excited by the THz magnetic near field with low pass filter. (b) The experiment data is excited by the THz pulse in free space without filter.

VI. Calculation of Faraday ellipticity angle

In this section, we derive theoretical expressions for the polarization ellipse of the NIR probe pulse after the propagation through the sample, which includes transmission through the crystal, reflection at the surface and again transmission through the crystal as shown in the inset (a) of Fig. S1.

The sublattice-magnetization dynamics, which can be presented by $\boldsymbol{m}_1 (z, t)$ and $\boldsymbol{m}_2 (z, t)$ at each depth position, are calculated by the Landau–Lifshitz–Gilbert (LLG) equation based on the two-lattice model:

$$\frac{d\boldsymbol{R}_i}{dt} = -\frac{\gamma}{(1+\alpha^2)}\left(\boldsymbol{R}_i \times [\boldsymbol{B}(z,t) + \boldsymbol{B}_{\text{eff},i}] - \alpha\boldsymbol{R}_i \times \left(\boldsymbol{R}_i \times [\boldsymbol{B}(z,t) + \boldsymbol{B}_{\text{eff},i}]\right)\right), \quad \text{(S6.1)}$$

where $\boldsymbol{R}_i = \boldsymbol{m}_i / m_0$ ($m_0 = |\boldsymbol{m}_i|$) is the unit vector of the sublattice magnetization, $\gamma = 1.76 \times 10^{11}$ s^{-1}T^{-1} is the gyromagnetic ratio, and $\boldsymbol{B}_{\text{eff},i}$ is the effective magnetic field determined by $-\partial V/\partial \boldsymbol{R}_i$ (i = 1,2), where V is the free energy of the iron spin system divided by m_0. The free energy can be written as

$$V = E\boldsymbol{R}_1 \cdot \boldsymbol{R}_2 + D(X_1 Z_2 - X_2 Z_1) - A_{xx}(X_1^2 + X_2^2) - A_{zz}(Z_1^2 + Z_2^2), \quad \text{(S6.2)}$$

where E and D are the symmetric and antisymmetric exchange fields, respectively, and A_{kk} is the anisotropy constant (k = x, z). X_i and Z_i are the projections of the vector $\boldsymbol{R}_i$ on the x- and z-axes, respectively. The second term in Eq. (S6.1) represents the magnetization damping with the Gilbert damping constant $\alpha = \alpha_0 + \alpha_1 \cdot |\Delta M_z/M_0|$. The magnetization dependent term $\alpha_1 \cdot |\Delta M_z/M_0|$ can be attributed to the four-magnon scattering process, which is thought to be the dominant microscopic spin scattering mechanism in a rare-earth ferrite [S1]. This term is considered to be the one of the reasons for the faster decay of the magnetization oscillation at stronger fields as shown in Fig. 4c.

To predict the ellipticity angle η measured in our experiment, the process of the propagation of the probe pulse through the sample is calculated. In this calculation, the 52-μm-thick crystal is divided into 260 cells along the z-axis, and the variation of polarization of probe pulse in each cell is calculated. In each cell, the magnetic field has been prepared by the FDTD simulation and the magnetization dynamics have been calculated by the LLG equation. The magnetization dynamics contribute to the permittivity tensor in each cell, and by using these permittivity tensors we calculate the propagation of the electric field of the probe light in $HoFeO_3$. We consider that the electric field of the probe pulse should obey the plane-wave equation along the z-axis in the sample [S2],

$$\mu\begin{pmatrix} \varepsilon_0 + \sigma & i\kappa \\ -i\kappa & \varepsilon_0 - \sigma \end{pmatrix}\frac{\partial^2 \boldsymbol{E}}{\partial t^2} = \frac{\partial^2 \boldsymbol{E}}{\partial z^2}, \quad \text{(S6.3)}$$

$$\kappa = f \cdot \frac{M_0 + \Delta M_z}{M_0} + g \cdot \frac{L_0 + \Delta L_x}{L_0}, \quad \text{(S6.4)}$$

where μ, ε_0, and σ are constants independent of the magnetization. ΔM_z is the temporal variation of the z-axis component of the ferromagnetic vector $\boldsymbol{M} = \boldsymbol{m}_1 + \boldsymbol{m}_2$, and ΔL_x is the temporal variation of the x-axis component of the antiferromagnetic vector $\boldsymbol{L} = \boldsymbol{m}_1 - \boldsymbol{m}_2$. M_0 and L_0 are the amplitudes of $\boldsymbol{M}$ and $\boldsymbol{L}$ at equilibrium. The phenomenological parameters f and g represent the contributions of ferromagnetic and anti-ferromagnetic vectors to the off-diagonal elements in Eq. (S6.3), respectively. Here, we ignored the symmetric terms in the off-diagonal elements which is the origin of the Cotton-Mouton effect, because this effect is negligible for the probe wavelength of 800 nm in the rare-earth orthoferrite [S3].

We consider that the electric field $\boldsymbol{E}$ of the probe pulse has the following form of a plane wave:

$$\boldsymbol{E} = \begin{pmatrix} E_x \\ E_y \end{pmatrix} e^{i(\omega t - kz)}. \quad \text{(S6.5)}$$

The normal modes of Eq. (S6.3) are

$$\boldsymbol{n}_+ = \begin{pmatrix} 1 \\ i(\Gamma - \sqrt{1+\Gamma^2}) \end{pmatrix} e^{i(\omega t - k_+ z)}, \tag{S6.6}$$

$$\boldsymbol{n}_- = \begin{pmatrix} 1 \\ i(\Gamma + \sqrt{1+\Gamma^2}) \end{pmatrix} e^{i(\omega t - k_- z)}, \tag{S6.7}$$

where k_+ and k_- are defined as

$$k_\pm = \sqrt{\omega^2 \mu(\varepsilon \pm \sqrt{\sigma^2 + \kappa^2})}, \tag{S6.8}$$

and $\Gamma = \sigma / \kappa$ indicates the relative strength of the birefringence with respect to the magneto-optic effect. Any electric field in the material can be expressed by a combination of the two normal modes, that is,

$$\boldsymbol{E}(z_0, t_0) = \alpha_+(z_0, t_0)\boldsymbol{n}_+ + \alpha_-(z_0, t_0)\boldsymbol{n}_-. \tag{S6.9}$$

After the propagation over a distance d, which is the length of one cell, the electric field becomes

$$\begin{aligned}\boldsymbol{E}(z_0 + d, t_0 + \tau) &= e^{i(\omega\tau - k_+ d)}\alpha_+(z_0, t_0)\boldsymbol{n}_+ + e^{i(\omega\tau - k_- d)}\alpha_-(z_0, t_0)\boldsymbol{n}_- \\ &\propto \alpha_+(z_0, t_0)\boldsymbol{n}_+ + e^{2i\delta d}\alpha_-(z_0, t_0)\boldsymbol{n}_-, \end{aligned} \tag{S6.10}$$

where $\delta = (k_+ - k_-)/2$ represents the wave-vector mismatch between the two normal modes. When the probe pulse reaches the $HoFeO_3$ surface with the microstructures, it is reflected by the air/$HoFeO_3$ interface and then passes through the sample again. The reflection of the electric field at the surface is described by

$$\begin{aligned}\boldsymbol{E}_r &= r_+\alpha_+\boldsymbol{n}_+ + r_-\alpha_-\boldsymbol{n}_- = \frac{1 - N_+}{1 + N_+}\alpha_+\boldsymbol{n}_+ - \frac{1 - N_-}{1 + N_-}\alpha_-\boldsymbol{n}_- \\ &\propto \alpha_+\boldsymbol{n}_+ + \frac{1 + N_+ - N_- - N_+N_-}{1 - N_+ + N_- - N_+N_-}\alpha_-\boldsymbol{n}_-, \end{aligned} \tag{S6.11}$$

where $N_\pm = ck_\pm / \omega$.

The values of the parameters used in our solution of LLG equation and calculation of magneto-optic effect are those reported in the literature [S4–6], and are summarized in the following Table I. We used the values of $YFeO_3$ as f and g [S6], because the magneto-optic effect in rare-earth orthoferrites is determined by electron transitions in Fe–O clusters and is influenced only slightly by the type of the rare-earth ion. We used the value Γ of 26 to obtain oscillation amplitudes in agreement with the experimental results, which is similar to the value of 32 in the literature [S5]. Here Finally, the obtained electric field $\boldsymbol{E}$ of the probe light is used to derive the major axis b_+ and minor axis b_- according to the following equation:

$$b_\pm = \sqrt{|E|^2 + 2Im(E_x^* E_y)} \pm \sqrt{|E|^2 - 2Im(E_x^* E_y)} \;. \tag{S6.12}$$

Supplementary Table 1| The parameters used in our calculation

Parameter	Value [S4–6]
E	640 T
D	14 T
A_{xx}	8.8×10^{-2} T
A_{zz}	1.9×10^{-2} T
α_0	2.27×10^{-4}
α_1	1×10^{-3}
Γ	26
δ	2.7×10^{5} rad/m
f	1×10^{-3}
g	$3 \times 10^{-3} \times e^{-i\frac{\pi}{4}}$

VII. Spin precession in a THz magnetic field

The spin motion caused by the THz magnetic field is explained as follows. The spin dynamics of antiferromagnets are described by the LLG equation provided in the Supplementary Information VI. If the damping term is ignored, the LLG equation reads

$$\frac{d\boldsymbol{m}_i}{dt} = -\gamma\left(\boldsymbol{m}_i \times [\boldsymbol{B}_{\mathrm{THz}} + \boldsymbol{B}_{\mathrm{eff},i}]\right), \qquad \text{(S7.1)}$$

where $\boldsymbol{m}_i$ (i = 1,2) represent the sub-lattice magnetizations, γ is the gyromagnetic ratio and $\boldsymbol{B}_{\mathrm{THz}}$ is the external THz magnetic field. $\boldsymbol{B}_{\mathrm{eff},i}$ is the effective magnetic field, which is determined by the magnetic potential of the antiferromagnet. Due to the existence of $\boldsymbol{B}_{\mathrm{eff},i}$, the spins have intrinsic precession modes at certain frequencies [S7]. Here, we only discuss the antiferromagnetic (AF) mode.

A precession is possible even without an external THz magnetic field: if $\boldsymbol{m}_i$ is perturbed away from the equilibrium position, the torque due to $\boldsymbol{B}_{\mathrm{eff},i}$ leads to an intrinsic precession as shown by the grey circle in the right panel of Fig. S8. From the LLG equation, we find that the direction of the torque due to $\boldsymbol{B}_{\mathrm{eff},i}$ is along the tangent of the grey circle, and the torque rotates at the frequency of the intrinsic

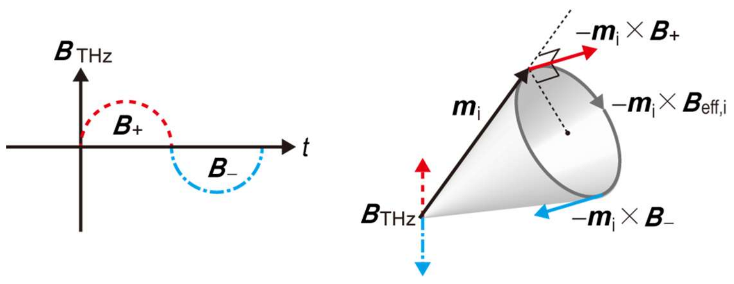

Figure S8. Precession of $\boldsymbol{m}_i$ in a THz magnetic field. Here, only one sub-lattice spin in the antiferromagnet is shown as an example.

spin precession.

If there is an additional THz magnetic field at the same frequency and phase as the intrinsic precession mode as shown in the left panel of Fig. S8, the torque due to $\boldsymbol{B}_{\mathrm{THz}}$ (red and blue solid lines in the right of Fig. S8) can always have the same direction as the torque due to $\boldsymbol{B}_{\mathrm{eff},i}$. Therefore, an oscillating magnetic field can resonantly excite the intrinsic spin precession.

VIII. Details of the motion of *L*

In the case of the static state, the canting angles of $\boldsymbol{m}_1$ and $\boldsymbol{m}_2$ with respect to the z axis are only ~0.6°. After strong excitation, the canting angles of $\boldsymbol{m}_1$ and $\boldsymbol{m}_2$ are in the range of 0.6±0.6°. Therefore, the motion of $\boldsymbol{m}_1$ and $\boldsymbol{m}_2$ is always restricted to the region around (a little higher than) the equator of the sphere $x^2 + y^2 + z^2 = |\boldsymbol{m}_1| = |\boldsymbol{m}_2|$. An exaggerated and strongly magnified image of the motions of $\boldsymbol{m}_1$ is shown in Fig. S9, and the projection of $\boldsymbol{m}_1$ in the xy plane represents the motion of $\boldsymbol{L}/2$. The motion of $\boldsymbol{L}$ can be described by different components on the x axis in the cases of the lower and upper semicircles. The difference of these components is defined by ΔX in the figure, and the ΔL_{x} mentioned in main article is also indicated in Fig. S9. Note that ΔX is relatively small ($\Delta X / \Delta L_{\mathrm{x}} \sim 0.002$), and thus it can be ignored.

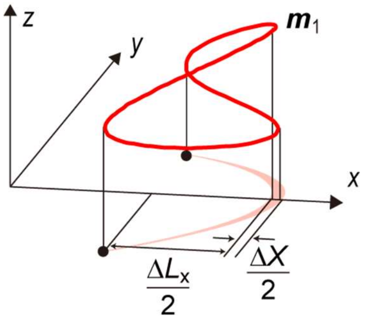

Figure S9. Schematic of the motion of $\boldsymbol{m}_1$.

The frequency-domain spectra of $\Delta M_z/M_0$ and $\Delta L_{\mathrm{x}}/L_0$ for $E_{\mathrm{THz}} = 0.8$ MV cm^{-1} are shown in Fig. S10 presented in the above response. Regarding the contributions of $\boldsymbol{M}$ and $\boldsymbol{L}$ to a single peak, we can identify two types of peaks: (1) For the 2ν and 4ν peaks, $\Delta M_z/M_0$ and $\Delta L_{\mathrm{x}}/L_0$ contribute similarly. (2) For the ν and 3ν peaks, the relative contribution of $\Delta L_{\mathrm{x}}/L_0$ is very small and its absolute magnitude lies close to or below the noise floor of our experiment.

The contribution of $\Delta L_{\mathrm{x}}/L_0$ to the fundamental peak can be understood by considering the motion of $\boldsymbol{m}_1$: An exaggerated and strongly magnified image of the motion of $\boldsymbol{m}_1$ is shown in Fig. S9, and the projection of $\boldsymbol{m}_1$ onto the x–y plane represents the sweep motion of $\boldsymbol{L}/2$. The frequency of this cycle of $\boldsymbol{m}_1$ is ν. During each cycle of $\boldsymbol{m}_1$, $\boldsymbol{L}/2$ sweeps two times across the x–y plane (from $+y$ to $-y$ and from $-y$ to $+y$), resulting in a frequency of 2ν with respect to the change ΔL_{x}. However, the two sweep

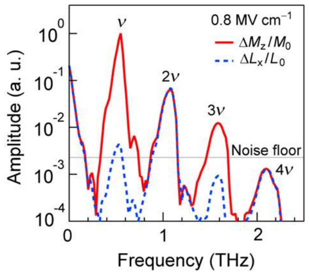

Figure S10. In the case of the 3rd harmonic, the peak amplitude of $\Delta L_{\mathrm{x}}/L_0$ is only 7.5% of that of $\Delta M_z/M_0$. The data in this graph is normalised to the fundamental peak amplitude of $\Delta M_z/M_0$ at frequency ν. The noise floor of our experiment is presented by the grey line. Note that "a.u." is the abbreviation for "arbitrary units".

trajectories of $\boldsymbol{L}/2$ do not completely overlap: the difference is shown by the shaded region in the x–y plane in Fig. S9. Because the difference $\Delta X/2$ only occurs one time for each cycle of $\boldsymbol{m}_1$, the ΔL_x oscillation at 2ν is slightly modulated by an oscillation at ν.

IX. Temporal variations of the fundamental, SH and TH peak areas without normalization

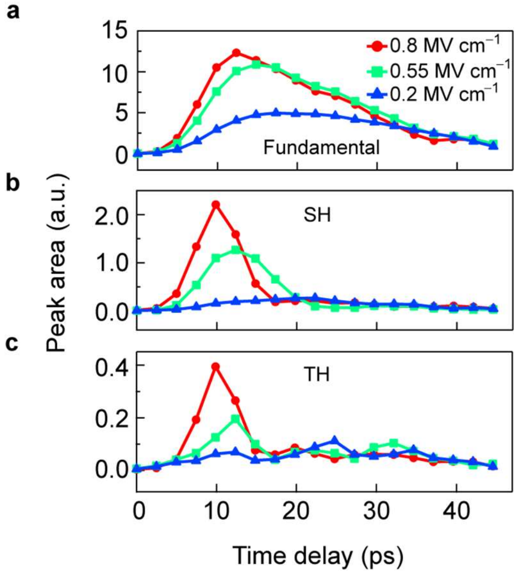

Figure S11. The temporal profiles without normalization for the fundamental, SH, and TH peak areas. (a) The fundamental, (b) SH, and (c) TH peak areas. The three figures are the data (circle: 0.8 MV cm^{-1}, square: 0.55 MV cm^{-1}, triangle: 0.2 MV cm^{-1}) shown in Fig. 4c but without normalization. Note that "a. u." is the abbreviation for "arbitrary units".

X. Time-integrated spectra of the magnetic field and the q-AF modes

The spectrum of the magnetic field at the center of the spiral is shown by the shaded area in Fig. S12. The peak is located at ν_c = 0.54 THz. The red and blue solid curves are the spectra of the

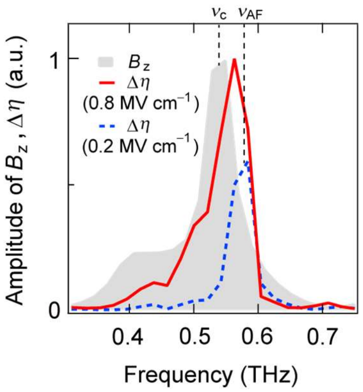

Figure S12. Spectra of the magnetic field and the q-AF modes. Note that "a.u." is the abbreviation for "arbitrary units".

experimentally obtained $\Delta\eta(t)$ extending over 47 ps for E_{THz} = 0.8 MV cm^{-1} and 0.2 MV cm^{-1}, respectively.

XI. Red shift of spin precession in a THz magnetic field

To see the relation of a strong damping with the change of resonance frequency, we performed simulations based on the LLG equation with different nonlinear damping constants α_1. As shown in Figs. S13a and S13b, the red shift becomes smaller as the damping increases. In other words, a stronger damping does not necessarily lead to a stronger redshift. The value of $\alpha_1 = 1 \times 10^{-3}$ is used for the calculation in our present work (see the Supplementary Information VI).

In addition, the red shift induced by the forced oscillation should not depend on the excitation amplitude. Therefore, we show here that the red shift is a result of the intrinsic nonlinear properties of the antiferromagnet in addition to the effect of forced oscillation in Figs. S13c and S13d. The intrinsic frequency of the antiferromagnetic (AF) mode can be extracted from the LLG equation to obtain the dependence on the amplitude of the spin precession. As the amplitude of the spin precession increases, the frequency of the AF mode decreases almost quadratically. This can also be verified by the LLG equation using different excitation amplitudes as shown in Fig. S13c.

Furthermore, we consider that the forced oscillation influences the results to some degree in this system. As shown in Figs. S13e and S13f, when the spin precession is excited non-resonantly by THz pulses at 0.47 THz, the spin precession frequency undergoes a red shift. When the spin precession is excited by THz pulses at 0.67 THz, the spin precession frequency undergoes a blue shift. When the spin precession is excited resonantly, the spin precession frequency still shows a red shift due to the nonlinearity discussed in the above paragraph.

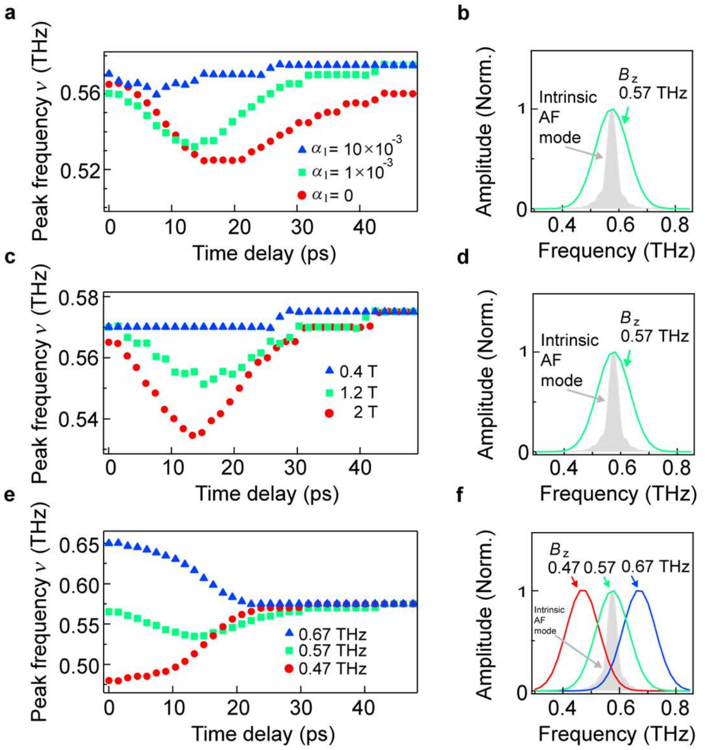

Figure S13. Calculation of red shift. (a) The theoretical red shift of the fundamental peak frequency for different nonlinear damping constants. (b) The input THz magnetic field has a peak at 0.57 THz, which is resonant with the antiferromagnetic (AF) mode. (c) The theoretical red shift of the fundamental peak frequency for different excitation amplitudes. (d) The input THz magnetic field is a Gaussian pulse with a peak frequency at 0.57 THz. (e) The theoretical red shift of fundamental peak frequency for different excitation frequencies. (f) The input THz magnetic fields are Gaussian pulses with peak frequencies at 0.47 THz, 0.57 THz and 0.67 THz, respectively.

XII. Data for longer decay times

To see the interference between oscillations with different frequencies clearly in our experimental results, the time-domain data for longer delay times are shown in Fig. S14 below. It can be confirmed that the spin precession completely decays after 120 ps. When the probe pulses are transmitted through the sample, they will feel different degrees of magnetization change because of the inhomogeneous distribution of the magnetic field along the *z* axis. As explained in the Supplementary Information XI, the frequency of spin precession shifts depending on the magnitude of the magnetization change. The interference among these components may contribute to the faster decay under stronger excitation conditions. Because the redshift increases with the excitation amplitude, the interference-induced decay should be faster in the case of stronger excitation.

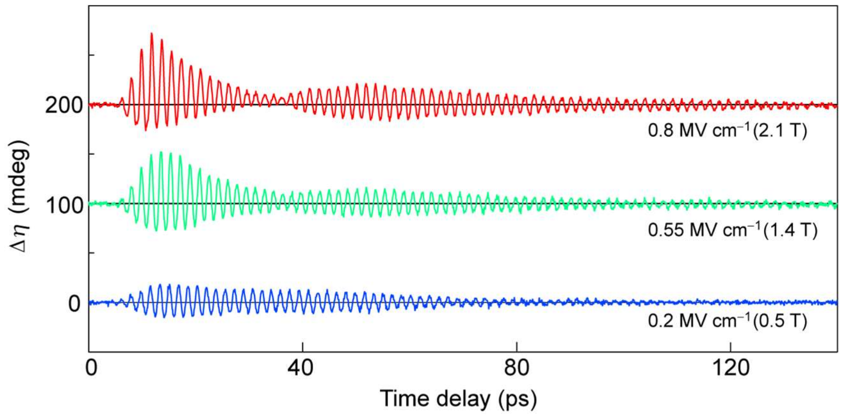

Figure S14. The data for longer delay times.

XIII. Beating effect on the Faraday signals

To explain the origin of the beating effect, we added calculation results to Fig. S15 below. The magnetic field distribution near the center of the spiral antenna is homogeneous with respect to the *x*–*y* plane. On the other hand, the distribution with respect to the depth direction (*z*-axis) is highly inhomogeneous as shown in Fig. S4 and also in Fig. S15a below. Since a magnetic field with a different amplitude excites a spin precession with a different red shift, the depth dependence of the magnetic field results in different precession motions that interfere when the probe pulse is transmitted through the sample.

A simple example is shown in Fig. S15b, where we consider the interference of $\Delta M_z/M_0$ components at four different depths. The considered *z* positions are marked by the arrows in Fig. S15a, and the

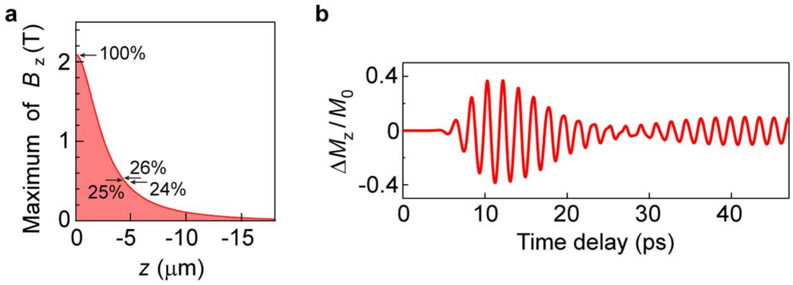

Figure S15. Origin of the beating signal. (a) Depth dependence of the maximum peak amplitude of the magnetic near-field in $HoFeO_3$. The $HoFeO_3$ surface including the microstructure is located at $z = 0$. (b) The average of the $\Delta M_z/M_0$ components at the four *z*-positions marked in (a).

corresponding magnetic field amplitudes are provided next to the arrows. As a result of the interference, a clear beating signal can be observed.

XIV. Field dependence for the fundamental, SH and TH peak areas

Fig. S16 shows normalized E_{THz} dependence of the fundamental peak area (a), that of the SH peak area (b) and that of the TH peak area (c). The data points are the peak areas obtained by integrating the corresponding Fourier transform amplitudes in the spectra. The grey lines are proportional to the first, second, and third power of E_{THz}. The error bars represent the noise floor in each spectrum.

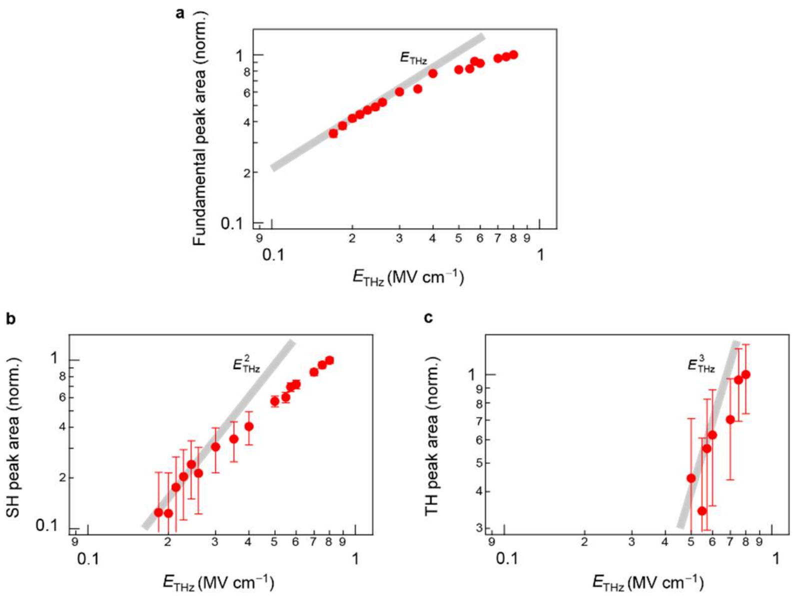

Figure S16. Field-strength dependence for the fundamental, SH and TH peak areas. (a), (b), (c), Normalized dependence of the fundamental (a), SH (b), and TH peak area (c) on the field strength E_{THz}. The error bars represent the noise floor in the spectra, which is obtained by integrating the Fourier transform amplitudes of the case for $E_{\mathrm{THz}} = 0.2$ MV cm^{-1} over the range from 1.25 THz to 1.71 THz.

XV. The selection rules of high harmonic generation of spin dynamics

In electric systems, the selection rules of high harmonic generation are usually discussed in terms of the nonlinear susceptibilities $\chi_{\mathrm{e}}^{(n)}$, which relate the electric field $\boldsymbol{E}$ and the electric polarization $\boldsymbol{P}$ through

$$\boldsymbol{P} = \varepsilon_0\chi_{\mathrm{e}}^{(1)}\boldsymbol{E} + \varepsilon_0\chi_{\mathrm{e}}^{(2)}\boldsymbol{EE} + \varepsilon_0\chi_{\mathrm{e}}^{(3)}\boldsymbol{EEE} + \cdots. \tag{S15.1}$$

The spatial inversion $\boldsymbol{r} \rightarrow -\boldsymbol{r}$ changes $\boldsymbol{P}$ to $-\boldsymbol{P}$ and $\boldsymbol{E}$ to $-\boldsymbol{E}$, whereas the nonlinear susceptibilities $\chi_{\mathrm{e}}^{(n)}$ stay unchanged:

$$-\boldsymbol{P} = -\varepsilon_0\chi_{\mathrm{e}}^{(1)}\boldsymbol{E} + \varepsilon_0\chi_{\mathrm{e}}^{(2)}\boldsymbol{EE} - \varepsilon_0\chi_{\mathrm{e}}^{(3)}\boldsymbol{EEE} + \cdots. \tag{S15.2}$$

From Eqs. (S15.1) and (S15.2) it can be inferred that $\chi_{\mathrm{e}}^{(n)} = 0$ for even orders (n = 2, 4, …) in the case of a system with inversion symmetry. The absence of the even-order susceptibilities translates into an absence of the even-order harmonics. A similar relation holds for the magnetic field $\boldsymbol{B}$ (= $\mu_0\boldsymbol{H}$) and the magnetization $\boldsymbol{M}$:

$$\mu_0 \boldsymbol{M} = \chi_{\mathrm{m}}^{(1)} \boldsymbol{B} + \chi_{\mathrm{m}}^{(2)} \boldsymbol{B}\boldsymbol{B} + \chi_{\mathrm{m}}^{(3)} \boldsymbol{B}\boldsymbol{B}\boldsymbol{B} + \cdots, \tag{S15.3}$$

where $\chi_{\mathrm{m}}^{(n)}$ denotes the nonlinear magnetic susceptibility. The fundamental principles of electromagnetism, that is, the Maxwell equations, tell us that a spatial inversion $\boldsymbol{r} \rightarrow -\boldsymbol{r}$ does not change the magnetic field $\boldsymbol{B}$ and the magnetization $\boldsymbol{M}$. The spatial inversion symmetry of a magnetic system thus imposes no constraint on the high harmonic generation spectra of spin dynamics, in sharp contrast to the behavior of electric systems. However, the magnetic susceptibilities $\chi_{\mathrm{m}}^{(n)}$ depend significantly on the magnetic order and the crystalline symmetry. Therefore, the selection rules of the high harmonic generation in magnetically ordered systems is not as trivial as in electric systems. To discuss the selection rules, we need to take a close look at the dynamical symmetry of $\chi_{\mathrm{m}}^{(n)}$ [S8,S9].

Fig. S17 shows two antiferromagentically ordered states in an AC magnetic field: (a) an antiferromagnetic (Néel) state with a dynamical symmetry and (b) a canted antiferromagnetic state without dynamical symmetry. We consider an AC magnetic field that periodically oscillates along the z axis as in our experiments, $B_z\cos(\Omega t)$. B_z and $\Omega = 2\pi/T$ denote the amplitude and the angular frequency of the magnetic field, respectively. We consider a spin rotation around the x axis in the presence of the AC magnetic field. The rotation angle is π, and this spin rotation acts on the sublattice magnetization components as follows: R_x^π: $\left(m_i^x(t), m_i^y(t), m_i^z(t)\right) \rightarrow \left(m_i^x(t), -m_i^y(t), -m_i^z(t)\right)$.

Since the AC magnetic field is coupled to $M^z = m_1^z + m_2^z$, the spin rotation R_x^π can be interpreted as a change of the orientation of the magnetic field from the viewpoint of the sublattice magnetization, $B_z\cos(\Omega t) \rightarrow -B_z\cos(\Omega t)$. This change can also be seen as a time shift equal to half of the magnetic-field oscillation period because

$$-B_z \cos(\Omega t) = B_z \cos\left[\Omega\left(t + \frac{T}{2}\right)\right]. \tag{S15.4}$$

Hence, the spin rotation R_x^π relates two different points in time, t and $t + T/2$. To derive the selection

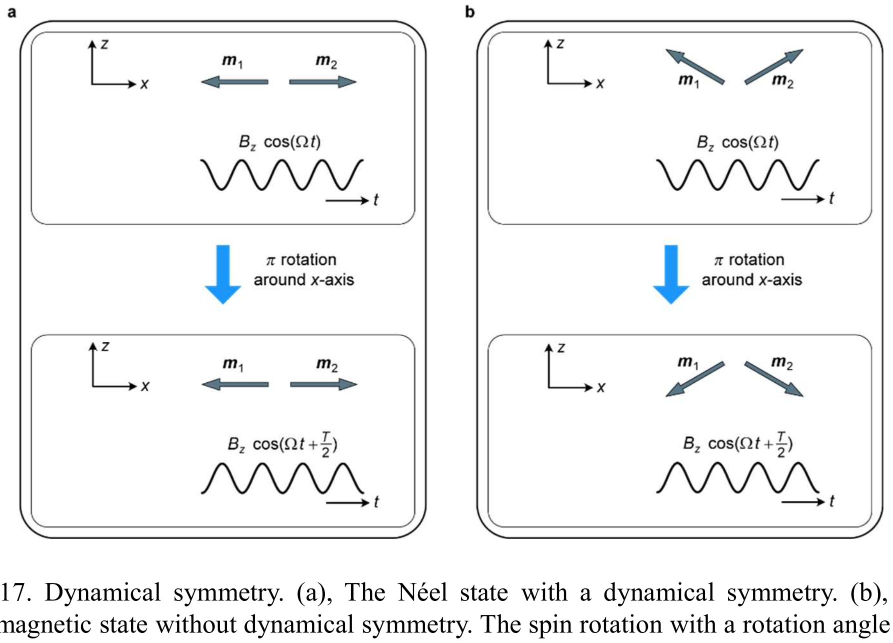

Figure S17. Dynamical symmetry. (a), The Néel state with a dynamical symmetry. (b), A canted antiferromagnetic state without dynamical symmetry. The spin rotation with a rotation angle of π does not change the Néel state while it changes the relative orientation of the periodically oscillating magnetic field. Thus, the Néel state has a dynamical symmetry according to Eq. (S15.5). On the other hand, the π rotation alters the canted antiferromagnetic state. Thus, the canted antiferromagnetic state has no dynamical symmetry.

rules, we need to link the magnetizations at these times. However, when we link the two sublattice magnetizations $\boldsymbol{m}_i(t)$ and $\boldsymbol{m}_i(t + T/2)$, we need to be aware of the initial configuration $\boldsymbol{m}_i(t = t_0)$ just before the magnetic field was turned on. For the Néel state in Fig. S17a, the initial configuration is invariant under the π rotation. This symmetry of the initial configuration leads to the following dynamical symmetry:

$$R_x^{\pi} m_i^z(t)(R_x^{\pi})^{-1} = -m_i^z\left(t + \frac{T}{2}\right). \tag{S15.5}$$

This dynamical symmetry implies $\tilde{m}_i^z(2n\Omega) = 0$ (n: integer), where $\tilde{m}_i^z(q\Omega) = \int_{-\infty}^{\infty} dt\, e^{iq\Omega}\, m_i^z(t)$ is the Fourier transform of $m_i^z(t)$. In other words, we found that the spectra cannot contain a second-order harmonic peak and only the third-order peak can appear. On the other hand, the canted state is not invariant under the π rotation around the x axis as shown in Fig. S17b. In other words, the canted state has no dynamical symmetry. The absence of the dynamical symmetry permits the coexistence of the second- and the third-order harmonics, in agreement with our experiments.

XVI. Symmetry of the magnetic potential

Fig. S18 shows how the magnetic potential [Eq. (S6.2)] is asymmetric due to the Dzyaloshinskii-Moriya interaction D when a z component of magnetic moment $Z_{1,2}$ emerges. From Fig. S18, one sees that when the system possesses a finite DM interaction D, the inversion symmetry $Z_{1,2} \rightarrow -Z_{1,2}$ (or the symmetry of π rotation in Sec. XV), is broken, i.e., $V(Z_{1,2}) \neq V(-Z_{1,2})$.

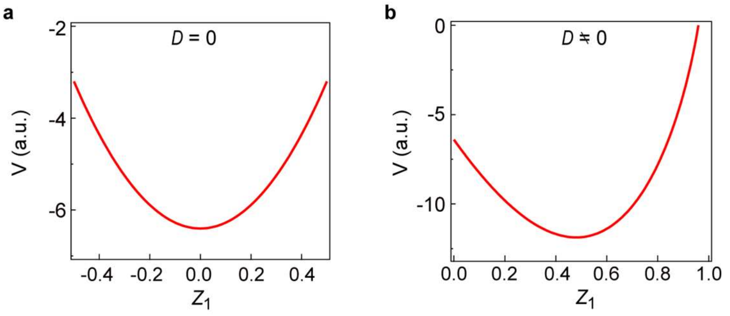

Figure S18. Magnetic potential as a function of Z_1 with/without the Dzyaloshinskii–Moriya interaction D. (a) In the case of D=0, the potential is a harmonic function with a minimum at Z_1=0. (b) In the case of $D \neq 0$, the potential is an anharmonic function with a minimum at $Z_1 \neq 0$. For this graph, we used a value of D that is much larger than the actual value for $HoFeO_3$. Note that "a.u." is the abbreviation for "arbitrary units", and the parameter Z_1 is dimensionless.

Supplementary References

S1. Tsang, C. H., White, R. L. & White, R. M. Spin-wave damping of domain walls in $YFeO_3$. *J. Appl. Phys.* **49**, 6063 (1978).

S2. Woodford, S. R., Bringer, A. & Blügel, S. Interpreting magnetization from Faraday rotation in birefringent magnetic media. *J. Appl. Phys.* **101**, 053912 (2007).

S3. Iida, R. et al. Spectral dependence of photoinduced spin precession in DyFeO3. *Phys. Rev. B.* **84**, 064402 (2011).

S4. Mukai, Y., Hirori, H., Yamamoto, T., Kageyama, H. & Tanaka, K. Nonlinear magnetization dynamics of antiferromagnetic spin resonance induced by intense terahertz magnetic field. *New J. Phys.* **18**, 013045 (2016).

S5. Tabor, W. J., Anderson, A. W. & Uitert, L. G. V. Visible and infrared Faraday rotation and birefringence of single-crystal rare-earth orthoferrites. *J. Appl. Phys.* **41**, 3018–3021 (1970)

S6. Zenkov, A. V. et al. Anisotropy of the Faraday effect in the weak ferromagnet $YFeO_3$. *Sov. Phys. JETP* **69**, 792 (1989).
S7. Herrmann, G. F. Resonance and high frequency susceptibility in canted antiferromagnetic substances. *J. Phys. Chem. Solid.* **24**, 597 (1963).
S8. Ikeda, T. N. & Sato, M. High-harmonic generation by electric polarization, spin current and magnetization. *Phys. Rev. B* **100**, 214424 (2019).
S9. Kanega, M., Ikeda, T. N. & Sato, M. Linear and nonlinear optical responses in Kitaev spin liquids. *Phys. Rev. Research* **3**, L032024 (2021).
